# Physical activity before and after breast cancer diagnosis and survival - the Norwegian women and cancer cohort study

**DOI:** 10.1186/s12885-015-1971-9

**Published:** 2015-12-16

**Authors:** Kristin Benjaminsen Borch, Tonje Braaten, Eiliv Lund, Elisabete Weiderpass

**Affiliations:** 1 Department of Community Medicine, Faculty of Health Sciences, University of Tromsø, The Arctic University of Norway, 9037 Tromsø, Norway; 2 Department of Research. Cancer Registry of Norway, Institute of Population-Based Cancer Research, 5313 Majorstuen, 0304 Oslo, Norway; 3 Department of Medical Epidemiology and Biostatistics, Karolinska Institutet, 171 77 Stockholm, Sweden; 4 Genetic Epidemiology Group, Folkhälsan Research Center, University of Helsinki, Biomedicum 1, Haartmansgatan 8, PB 63, FI-00014 Helsinki, Finland

**Keywords:** Breast cancer, Physical activity, Survival, Cohort

## Abstract

**Background:**

The main aim of this study was to investigate pre- and post-diagnostic physical activity (PA) levels, as well as changes in pre- and post-diagnostic PA levels, and their association with all-cause and breast cancer-specific mortality in women with breast cancer. Our study will add to the knowledge on whether a modifiable behavior such as PA can improve survival.

**Methods:**

We included 1,327 women with breast cancer from the population-based Norwegian Women and Cancer study, which enrolled women from 1991 to 2003. Breast cancer cases were identified through linkage to the Cancer Registry of Norway; date and cause of death were obtained from the National Register for Causes of Death through 31 December 2012. Self-reported pre- and post-diagnostic PA levels were assessed, and Cox proportional hazard regression and spline regression were used to evaluate the associations.

**Results:**

Pre-diagnostic PA levels were not associated with all-cause or breast cancer-specific mortality. Post-diagnostic PA levels were associated with a significant trend (*P <* 0.001) of decreased all-cause and breast cancer-specific mortality, which was stronger among older women (aged 50–74 years) and did not differ across categories of body mass index. All-cause mortality (hazard ratio [HR] = 1.76, 95 % confidence interval [CI] 1.21–2.56) and breast cancer-specific mortality (HR = 2.05, 95 % CI 1.35–3.10) increased among women who reduced their post-diagnostic PA level. These values were similar among women whose maintained an inactive PA level pre- and post-diagnosis.

**Conclusion:**

Overall, we observed a dose–response trend, with an inverse association between increased post-diagnostic PA level and all-cause and breast cancer-specific mortality, as well as a higher mortality risk among women who reduced their post-diagnostic PA levels. Our results are very promising for women with breast cancer, and indicate that health care professionals should consider adding PA as a part of primary cancer treatment.

**Electronic supplementary material:**

The online version of this article (doi:10.1186/s12885-015-1971-9) contains supplementary material, which is available to authorized users.

## Background

Breast cancer is the most common cancer among women worldwide, accounting for 25 % of all new cancers [1]. Despite advances in early detection and treatment, which have improved survival, breast cancer is the second most frequent cause of cancer death among women in most countries [1]. In the period 2008–2012 in Norway, the 5-year relative survival for patients diagnosed with breast cancer was 89 % [2].

Important research has revealed evidence that physical activity (PA) can help relieve treatment-related symptoms and improve quality of life and physical functioning [3, 4] in breast cancer survivors, whose numbers are increasing. Moreover, PA may decrease breast cancer recurrence and extend overall breast cancer survival and disease-free breast cancer survival [5–8]. However, in their recently released report, the Continuous Update Project of the World Cancer Research Fund concluded that there is limited evidence for an association between pre-diagnostic and post-diagnostic PA levels and all-cause and breast cancer-specific mortality among women with breast cancer [9]. A recent meta-analysis included 49,095 breast cancer survivors and reported a 23 % decreased relative risk of all-cause and breast cancer-specific mortality with increased PA levels [6]. Data from the Breast Cancer Pooling Project indicated that engagement in at least 10 metabolic equivalent (MET)-h/week (i.e., walking 2 miles in 30 min) of PA was associated with a 27 % reduction in all-cause mortality and a 25 % reduction in breast cancer-specific mortality among breast cancer survivors [10]. However, changes in pre- and post-diagnostic PA levels and their association with breast cancer survival have so far received limited attention [11–13]. Furthermore, it is import to understand whether increasing PA after cancer diagnosis is beneficial for breast cancer survivors, and if increased post-diagnostic PA can in fact improve survival.

In the present paper, we aimed to investigate pre- and post-diagnostic PA levels, as well as changes in pre- and post-diagnostic PA levels, and their association with all-cause mortality and breast cancer-specific mortality in a population-based sample of women with breast cancer in Norway.

## Methods

### Study sample

We used data from a national cohort of 109,398 women enrolled in the Norwegian Women and Cancer (NOWAC) study between 1991 and 2003. The NOWAC study is a prospective cohort study with a random sample of women aged 30–70 years drawn from the National Population Register in Norway [14, 15]. Women in the NOWAC study were linked to various population-based registries using the unique, 11-digit, national personal identification number.

Women in the NOWAC study completed an extensive questionnaire, including questions on PA level, height and weight, exogenous hormone use, previous illnesses, smoking status and habits, alcohol consumption, education, reproductive history, and dietary habits at enrollment, and again at two separate follow-up periods. Therefore, the present study had prospective data on PA at enrollment and at follow-up.

Women diagnosed with a primary, invasive, malignant neoplasm of the breast based on the 10^th^ revision of the International Statistical Classification of Diseases, Injuries and Causes of Death (ICD-10) codes C50.0–50.9 [16] were identified through record linkage to the Cancer Registry of Norway, from which date of diagnosis and tumor stage (by the pTNM system) were obtained. Information on date of death or emigration was obtained through record linkage to the Norwegian National Population Register. Information on cause of death was obtained through record linkage to the National Register for Causes of Death, in which physician-assigned ICD-10 codes for cause of death are given. We categorized these causes into all-causes combined (i.e., all-cause mortality) and breast cancer-specific mortality. Follow-up time was defined as the interval between the date of completion of the first follow-up questionnaire after breast cancer diagnosis and date of death from breast cancer, date of death from any other cause, date of emigration, or 31 December 2012 (last complete follow-up date), whichever came first.

We identified 3,867 women with a diagnosis of breast cancer recorded in Cancer Registry of Norway during the study period. We excluded 2,540 women due to missing information on PA levels, 437 of whom died before the second follow-up. This left 1,327 women with information on PA levels at enrollment and at follow-up in the final study sample. To evaluate the representativeness of the study sample, we compared selected characteristics at enrollment of excluded women and of our study sample. The Regional Ethical Committee and the Norwegian Data Inspectorate approved the NOWAC study. All women gave written informed consent prior to their participation in the NOWAC study.

### Assessment of physical activity and covariates

PA was defined in the NOWAC study questionnaire as follows: “By physical activity we mean activity both at work and outside work, at home, as well as training/exercise and other physical activity, such as walking, etc. Please mark the number that best describes your level of physical activity; 1 being very low and 10 being very high”. Thus PA level was assessed by self-report on an ordinal scale of 1 to 10. This PA scale has been validated [17], and refers to the total amount of PA across different domains, including recreation, occupation, transportation, and household in one global score. Moderate, but significant (*P <* 0.001) Spearman’s rank correlation coefficients were found (range: 0.36–0.46) between PA level at enrollment and concurrent outcomes from criterion measures of a combined sensor monitoring heart rate and movement. The scale ranged from 1 (very low) to 10 (very high), and corresponded to mean values of 0.8 and 3.4 h/day of moderate/vigorous PA, respectively, with a linear increase (P for trend <0.001), and appeared valid to rank PA level in Norwegian women, but not to quantify a definite dose of PA [17].

Pre-diagnostic PA level in the present analysis refers to PA level at enrollment. Post-diagnostic PA level refers to the PA level reported in first follow-up questionnaire completed after breast cancer diagnosis.

Information on covariates was also obtained from the NOWAC study questionnaire. Information on height and weight was used to calculate body mass index (BMI, weight (kg)/squared height (m)). Time from breast cancer diagnosis to post-diagnostic PA assessment was calculated as the number of days between diagnosis and the completion of the applicable follow-up questionnaire.

### Statistical analyses

Descriptive statistics (means and standard deviations (SD)) were conducted. Multivariable Cox proportional hazard models were used to estimate hazard ratios (HR) and 95 % confidence intervals (CI). Tests for homogeneity were conducted using Wald statistics. Analyses included the association of pre- and post-diagnostic PA level and changes in pre- and post-diagnostic PA levels with the two outcomes of interest: all-cause mortality and breast cancer-specific mortality.

When analyzing the association between pre-diagnostic and post-diagnostic PA levels and all-cause and breast cancer-specific mortality, the 10 levels of the PA scale were collapsed into 5 as follows: 1–2, 3–4, 5–6, 7–8, and 9–10. In analyses of the change in pre- and post-diagnostic PA levels, a PA level ≥5 was considered active; we defined reduced PA level as a pre-diagnostic PA level ≥5 and post-diagnostic PA level <5; women with pre- and post-diagnostic PA levels <5 were categorized as maintained inactive; increased PA level was defined as a pre-diagnostic PA level <5 and a post-diagnostic PA level ≥5; and women with pre- and post-diagnostic PA levels ≥5 were categorized as maintained active. The multivariable models were adjusted for age at diagnosis, tumor stage (I, II, III, IV and unknown), pre-diagnostic PA levels (in models with post-diagnostic PA as the exposure) and number of days between breast cancer diagnosis and post-diagnostic PA assessment (≤365/>365 days). Additional analyses were conducted separately by age at diagnosis (34–49 and 50–74 years) and post-diagnostic BMI (<25 and ≥25 kg/m^2^). Stratified analyses for change in pre- and post-diagnostic PA levels were also done in subgroups of change in pre-and post-diagnostic BMI, defined as unchanged <25 kg/m^2^, weight gain (from BMI <25 to ≥25 kg/m^2^), weight loss (from ≥25 to <25 kg/m^2^) and unchanged ≥25 kg/m^2^. However, as we did not have the study power necessary to demonstrate statistically significant differences due of the small number of events in some of the subgroups, these results are not reported. We also carried out analyses that excluded women with a post-diagnostic PA assessment ≤365 days after breast cancer diagnosis to avoid bias due to possible declining PA prior to and after breast cancer diagnosis. Sensitivity analyses were carried out including other covariates, such as hormonal therapy (menopausal) use (ever/never), and comorbidities, such as diabetes and cardiovascular diseases, smoking status (ever/never), pack-years smoked, alcohol consumption (g/day), and duration of education (years). However, as these covariates had no statistically significant impact on the investigated associations, these analyses are not reported in the main results. Possible departures from linearity in the association between pre- and post-diagnostic PA levels and all-cause mortality were assessed using cubic spline regression [18]. The proportional hazard assumption was checked using Schoenfeld residuals and Kaplan-Meier plots, which suggested no evidence of deviation from proportionality. All the analyses were performed using the statistical package STATA, version 13, with all statistical tests two-sided and conducted at the 0.05 significance level.

## Results

Mean follow-up time in the study sample was 10.6 years (range 0.92–21.5). In total 197 women died during follow-up. The causes of death were: breast cancer (78.7 %), other cancers (13.2 %), and other causes (8.1 %) (Table 1). At the end of follow-up, 1,130 women were still alive, two of whom had emigrated and were censored at the time of emigration. The women in the study sample were younger than excluded women at breast cancer diagnosis (53.3 versus 59.3 years), had a slightly longer duration of education (1 year), fewer women in the study sample were current smokers (4 %), and alcohol consumption in the study sample was somewhat higher than that among excluded women (3.98 versus 3.28 g/day) (data not shown). There was only a negligible difference in PA at enrollment between included and excluded women (5.6 versus 5.5) (data not shown). A higher proportion of women who were excluded due to missing post-diagnostic PA assessment had stage I breast tumors (data not shown). Most women (90.6 %) in the study sample were postmenopausal at breast cancer diagnosis and mean age at death was 60 years. There was no statistically significant difference in age at diagnosis among women with different PA levels. Moreover, there were no differences in pre-diagnostic PA levels between women who were diagnosed shortly after enrollment and those diagnosed several years after enrollment, or in post-diagnostic PA levels by time from diagnosis to follow-up questionnaire (data not shown). Post-diagnostic PA level was not significantly different among women with different tumor stages at diagnosis (data not shown). The mean time between the date of pre-diagnostic PA assessment and date of diagnosis was 6.6 years (SD 3.87), while the mean time from the date of diagnosis to the post-diagnostic PA assessment was 3.08 years (SD 2.07).

**Table 1:** Selected characteristic of 1,327 women diagnosed with breast cancer in the Norwegian Women and Cancer study with complete information on pre- and post-diagnostic physical activity (PA) levels, 1991-2011

Characteristic	
Age at enrollment, mean (SD)	46.7 (7.0)
Age at breast cancer diagnosis, mean (SD)	53.3 (6.7)
Duration of mean follow-up, years (range)	10.6 (0.92–21.5)
Tumor stage, n (%)	
I	591 (44.5)
II	534 (40.2)
III	25 (1.8)
IV	20 (1.5)
Unknown	158 (11.9)
Cause of death, n (%)	
Breast cancer	155 (78.7)
Other cancers	26 (13.2)
Other causes	16 (8.1)
Postmenopausal at diagnosis (%)	1,202 (90.6)
Pre-diagnostic PA, mean (SD)	5.6 (1.85)
Post-diagnostic PA, mean (SD)	5.5 (1.83)
BMI (kg/m^2^) at enrollment, mean (SD)	23.6 (3.7)
BMI (kg/m^2^) after breast cancer diagnosis, mean (SD)	25.2 (4.0)
Hormone therapy use at enrollment (ever), n (%)	338 (23.8)
Comorbidities at enrollment, n (%)	
Diabetes	16 (1.1)
Cardiovascular diseases	58 (4.1)
Smoking status at enrollment, n (%)	
Current	421 (31.9)
Former	436 (33.1)
Pack-years smoked at enrollment (ever), mean (SD)	8.7 (7.7)
Alcohol consumption (g/day) at enrollment, mean (SD)	3.98 (5.0)
Education (years) at enrollment, mean (SD)	12.7 (3.5)

*SD* standard deviation, *BMI* body mass index

**Fig. 1:**
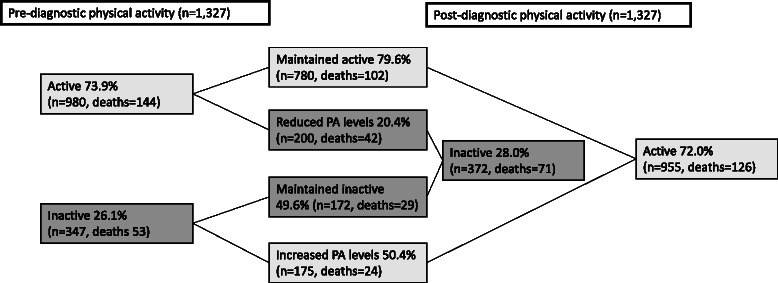
Changes in pre- and post-diagnostic physical activity (PA) levels of 1,327 women diagnosed with breast cancer in the Norwegian Women and Cancer study. Inactive = PA level 1–4, Active = PA level 5–10. Reduced PA level = pre-diagnostic PA level ≥5 and post-diagnostic PA level <5, Maintained inactive = pre- and post-diagnostic PA levels <5, Increased PA level = pre-diagnostic PA level <5 and a post-diagnostic PA level ≥5, Maintained active = pre- and post-diagnostic PA level ≥5

Nine hundred eighty women had a pre-diagnostic PA level of active (PA level ≥5); 79.6 % maintained this PA level post-diagnosis, whereas the rest had reduced PA levels (Fig. 1). Half of the women who reported pre-diagnostic PA levels of inactive had increased PA levels. The result was a distribution of post-diagnostic PA levels that was nearly identical to that of the pre-diagnostic PA levels, with 28 % of women classified as inactive and 72 % as active (Fig. 1). More than 80 % of the women who changed their PA levels moved only 1–2 levels on the PA scale. Pre- and post-diagnostic BMI was inversely associated (*P <* 0.001) with post-diagnostic PA level (i.e., the lower the pre- or post-diagnostic BMI the higher the post-diagnostic PA level) (data not shown). Overall, there was a significant increase in mean post-diagnostic BMI compared to pre-diagnostic BMI, indicating that the women gained weight after breast cancer diagnosis, as no differences in height were observed (Table 1). The increase in BMI observed in our study sample was similar to that seen during follow-up in the full NOWAC study cohort (data not shown).

### Pre-diagnostic physical activity level

**Fig. 2:**
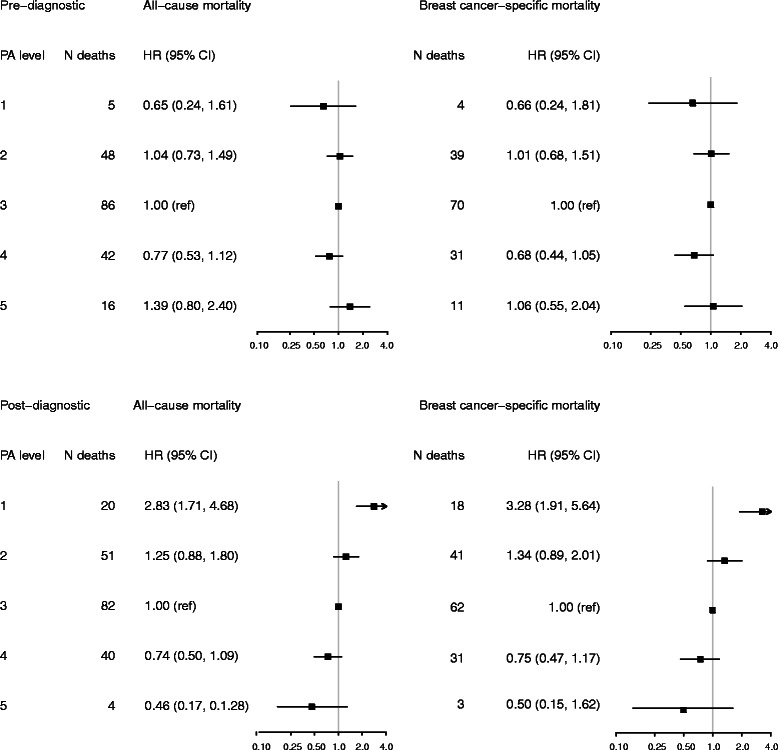
Hazard ratios (HR) and 95 % confidence intervals (CIs) of all-cause and breast cancer-specific mortality according to pre-diagnostic and post-diagnostic physical activity (PA) levels among 1,327 women from the Norwegian Women and Cancer study, 1991–2011. Multivariable model for pre-diagnostic PA adjusted for age and tumor stage and for post-diagnostic PA level adjusted for age, tumor stage, pre-diagnostic PA level and time from diagnosis to post-diagnostic PA assessment ≤365/>365 days

Pre-diagnostic PA level was not associated with all-cause or breast cancer-specific mortality (Fig. 2). The HR estimates did not change significantly when BMI, use of hormone therapy, or smoking status were added to the model, nor when stratified by BMI (<25 or ≥25 kg/m^2^) (data not shown).

### Post-diagnostic physical activity level

**Fig. 3:**
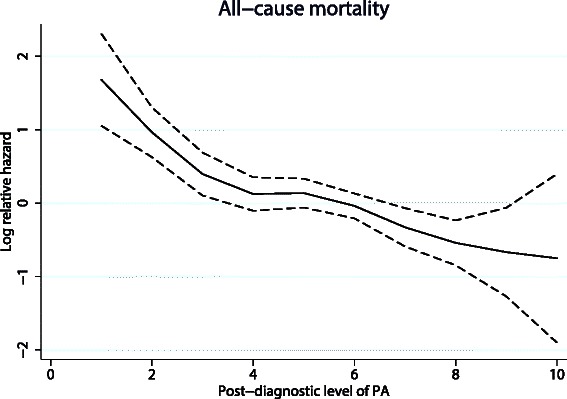
Log relative hazard with 95 % confidence intervals of all-cause mortality by post-diagnostic physical activity (PA) level using cubic regression splines

There was a statistically significant trend of decreasing all-cause mortality with increasing post-diagnostic PA levels (*P <* 0.001). Compared to women with a PA level of 5–6, women with a PA level of 1–2 had an almost three-fold increased all-cause mortality risk (HR = 2.83, 95 % CI 1.71–4.68), while women with a PA levels of 9–10 had a HR of 0.46 (95 % CI 0.17–1.28) (Fig. 2). The findings were similar for breast cancer-specific mortality (Fig. 2). These results were consistent across sensitivity analyses adjusted for age, tumor stage, pre-diagnostic PA level, and time from breast cancer diagnosis to post-diagnostic PA assessment; and also when use of hormone therapy, BMI, and smoking were added to the multivariable models (data not shown). Despite significant trends in the association between post-diagnostic PA level and all-cause mortality, the spline regression analysis indicated that the association was stronger for PA levels <5, while the association for PA levels ≥5 did not reveal a similar steep slope (Fig. 3). Analyses stratified by BMI (<25 or ≥ 25 kg/m^2^) indicated similar, statistically significant trends of reduced all-cause and breast cancer-specific mortality with increasing post-diagnostic PA levels for women with a BMI <25 kg/m^2^ (Additional file 1). This was true also for women with BMI ≥25 kg/m^2^ and low levels of post-diagnostic PA (Additional file 1), whereas women with a BMI ≥25 kg/m^2^ and the highest level of PA seemed to have an increased risk, though it was not significant. Reduced all-cause mortality related to post-diagnostic PA level was observed in women younger than 50 years of age (P for trend = 0.030*)* and those aged 50 years or older (P for trend <0.001); however, among women with a PA level of 1, the mortality risk was significantly stronger among women aged 50 years or older compared to those younger than 50 years of age (Additional file 2). The results were consistent for breast cancer-specific mortality (Additional file 2).

### Changes in pre- and post-diagnostic physical activity levels

Compared to women who maintained a PA level of active, all-cause mortality (HR = 1.76, 95 % CI 1.21–2.56) and breast cancer-specific mortality (HR = 2.05, 95 % CI 1.35–3.10) were both statistically significantly increased among women with reduced PA levels. This was also true for women who maintained a PA level of inactive compared to those who maintained a PA level of active, though this result was not significant (Table 2). Women with increased PA levels had a higher risk of all-cause and breast cancer-specific mortality, but this risk was not statistically significant (Table 2). The results were consistent across models adjusted for BMI (data not shown).

**Table 2:** Hazard ^b^ratios (HR) and 95 % confidence intervals (CIs) by all-cause mortality^a^ and breast cancer-specific mortality^b^ and change in pre- to post-diagnostic PA level among 1,327 women from the Norwegian Women and Cancer study, 1991–2011

Changes in PA level	N deaths	All-cause mortality HR (95 % CI)^c^	N deaths	Breast cancer-specific mortality HR (95 % CI)^c^
Maintained inactive	29	1.74 (1.00, 3.05)	23	1.66 (0.88, 3.12)
Reduced PA	42	1.76 (1.21, 2.56)	36	2.05 (1.35, 3.10)
Maintained active	102	1.00 (ref)	76	1.00 (ref)
Increased PA	24	1.23 (0.69, 2.18)	20	1.20 (0.63, 2.28)

^a^All causes of death combined

^**b**^Breast cancer as underlying cause of death

^c^Multivariable model adjusted for age, tumor stage at diagnosis and time from diagnosis to PA assessment ≤365/>365 days

## Discussion

In this prospective study of women with breast cancer, pre-diagnostic PA levels had no impact on all-cause and breast cancer-specific mortality, regardless of body size or age at diagnosis. Conversely, post-diagnostic PA levels decreased all-cause and breast cancer-specific mortality significantly in a dose–response manner, and these findings were consistent across different BMI groups. However, all-cause mortality was considerably increased among women with a PA level of 1–2 (on a scale of 10) who were older than 50 years as compared to younger women with the same PA level. Women with breast cancer who had reduced PA levels or maintained an inactive PA level had a substantial increase in all-cause mortality and breast cancer-specific mortality as compared to those who maintained an active PA level (i.e. ≥5).

### Pre-diagnostic physical activity level and all-cause and breast cancer-specific mortality

Findings from a recent review by Schmid and Leitzmann [6] indicated a decreased risk of all-cause (HR = 0.77, 95 % CI 0.66–0.90) and breast cancer-specific (HR = 0.77, 95 % CI 0.69–0.88) mortality associated with pre-diagnostic PA level, whereas our findings showed no such association. However, only two of the studies [12, 19] included in the meta-analysis assessed total PA and all-cause and breast cancer-specific mortality; the remainder of the studies reported only recreational PA [6]. In the recent, updated meta-analysis from the Continuous Update Project of the World Cancer Research Fund [20], which includes the same two studies [12, 19], total pre-diagnostic PA level showed an inverse, non-significant association with all-cause mortality (HR = 0.83, 95 % CI 0.62–1.12), with similar findings for breast cancer-specific mortality (HR = 0.80, 95 % CI 0.59–1.10). For recreational PA, the meta-analysis of the Continuous Update Project included eight studies on all-cause mortality and seven studies on breast cancer-specific mortality; their findings indicated a significant reduction in all-cause and breast cancer-specific mortality (26 % and 24 %, respectively) [20]. However, there was heterogeneity between the included studies, especially related to the methods used to measure PA, which precluded analyses of dose–response in the meta-analysis.

Our results did not change when analyses were stratified by BMI. Other studies have found significant associations between recreational pre-diagnostic PA level and all-cause mortality in women with a pre-diagnostic BMI <25 kg/m^2^, but not for women with a pre-diagnostic BMI ≥25 kg/m^2^ [21], while the opposite was found in a study by Abrahamson and colleagues [22]. Cleveland and colleagues [23] reported that recreational pre-diagnostic PA level was inversely related to all-cause mortality, independent of BMI.

### Post-diagnostic physical activity level and all-cause and breast cancer-specific mortality

Our analysis showed a significant trend of decreased all-cause and breast cancer-specific mortality associated with post-diagnostic PA levels ≥5. Of three published studies investigating total post-diagnostic PA, only the Health, Eating, Activity and Lifestyle (HEAL) study [12] reported a significant reduction in all-cause mortality related to total post-diagnostic PA level; the other two studies found an inverse relationship, but it was not statistically significant [13, 24]. The findings of the latest meta-analysis, which included these three studies, confirmed an inverse relationship for all-cause mortality (HR = 0.63, 95 % CI 0.41–0.97), but this relationship was not statistically significant for breast cancer-specific mortality (HR = 0.80, 95 % CI 0.59–1.10) and no dose–response relationship was observed [20]. Furthermore, the same meta-analysis for recreational post-diagnostic PA reported a reduced breast cancer mortality of 24 % [20]. Although the relationship between recreational post-diagnostic PA level and all-cause mortality appears to be stronger, studies on this topic are heterogeneous [20]; therefore extra caution must be taken when interpreting their results. In the Women’s Health Initiative Cohort (WHI), Irwin and colleagues [11] observed a 46 % reduction in all-cause mortality and a 39 % reduction in breast cancer-specific mortality when comparing women with the lowest versus the highest recreational PA levels. The Long Island Breast Cancer study [25] investigated recreational post-diagnostic PA and survival after breast cancer diagnosis and found that, compared to inactive women, women who were highly active (>9.0 MET h/week) after diagnosis reduced their all-cause mortality (HR = 0.3, 95 % CI 0.22–0.48) and their breast cancer-specific mortality (HR = 0.27, 95 % CI 0.15–0.46).

In our study there was no significant difference in the relationship between post-diagnostic PA levels and all-cause mortality and breast cancer-specific mortality for women with BMI <25 kg/m^2^, however for women with a BMI ≥25 kg/m^2^ and the highest post-diagnostic PA levels, all-cause and breast cancer-specific mortality increased. These results should be interpreted with caution, as there were a small number of events in some of the subgroups, and the test for homogeneity was not significant. Other studies reported no difference in the impact of post-diagnostic PA level by BMI, menopausal status, time since diagnosis, or tumor hormone receptor status [6, 25].

We found that, compared to women below 50 years of age, women aged 50 years or over at breast cancer diagnosis had a higher risk of all-cause and breast cancer-specific mortality if they reported very low post-diagnostic PA levels. Thus, PA seems to be of particular importance among older women, since their overall mortality risk is already higher due to age and risk of other comorbidities. Older breast cancer patients are a vulnerable group due to decreased physical functioning, and PA could play an important role in sustaining functional mobility [26]. Most importantly, our study revealed a potential decreased all-cause and breast cancer-specific mortality among women with breast cancer that had post-diagnostic PA levels of at least 5. PA is a modifiable behavior, which can to a large extent be controlled by women with breast cancer.

### Changes in pre- and post-diagnostic physical activity levels and all-cause and breast cancer-specific mortality

To our knowledge, there are few studies on the relationship between changes in pre- and post-diagnostic PA levels and all-cause and breast cancer-specific mortality [11–13]. The WHI study reported a significant, reduced all-cause mortality of 33 % when recreational PA increased from before to after breast cancer diagnosis, but no associations were found when PA decreased after diagnosis.[11]. The HEAL study reported significant, increased all-cause mortality with decreasing PA [12]. However, neither study could confirm a significant reduction in breast cancer-specific mortality [11, 12]. The Women’s Healthy Eating and Living study found no association between change in PA level post-diagnosis and all-cause mortality [13]. Our findings for all-cause mortality and breast cancer-specific mortality revealed that maintaining an inactive PA level or reducing one’s PA level after breast cancer diagnosis can lead to a higher mortality risk, even if we failed to demonstrate that increasing PA levels yielded a lower risk. Also, it seems that a change of one or two levels on the PA scale is sufficient to drive the associations, indicating that small changes are of importance. A substantial proportion of women in our study who were active before breast cancer diagnosis reported lower PA levels after diagnosis. It was beyond the scope of our study to investigate the reasons for this decrease, but it reveals a potential for interventions aiming to decrease all-cause and breast cancer-specific mortality.

## Strengths and limitations

Study strengths include a population-based cohort, with complete assessment of breast cancer incidence and death, taking advantage of the population-based cancer and death registries in Norway. Moreover, PA was assessed prospectively before diagnosis. The PA scale used in this study has been validated through moderate correlations that are considered sufficient to differentiate between different PA levels in a population of Norwegian women [17]. However, the PA assessment may not apply to women in other countries. The PA assessment in this study comprised all areas of PA, not only recreational PA. However, we are aware that a total self-reported measure cannot differentiate intensity, duration, and frequency of PA, nor the type of PA. A recently published study by de Glas and colleagues suggested that the intensity of PA is not related to the association between PA and improved overall survival in women with breast cancer [26].

Study limitations include the absence of information on breast cancer treatment. However, we did adjust for tumor stage at diagnosis. In Norway, cancer treatment follows national guidelines by tumor stage, thus, stage can be considered a valid surrogate variable for breast cancer treatment, with rare exceptions. Moreover, in the HEAL study, which adjusted for tumor stage, further adjustment for treatment did not change the HR [12]. Furthermore, it was not possible to perform subgroup analysis by tumor receptor status in our study due to the small number of cases in each subgroup. As most of the women were postmenopausal at cancer diagnosis, we could not perform subgroup analysis by menopausal status. We performed analyses including several variables that could be considered potential confounders in the association between PA and breast cancer risk, such as BMI, use of hormone therapy, other comorbidities, education, smoking, and alcohol consumption. However, as the inclusion of these variables did not change the HR estimates, we omitted these in the final analysis. Moreover, the potential for residual confounding remains. Measurement errors in self-reported information cannot be ruled out; however such an error would likely lead to a non-differential bias and the potential underestimation of the true effect. If breast cancer patients had been less physically active due to symptoms of the disease at the time of PA assessment, the bias of reversed causality could threaten the results. However, we did not discover any differences in the estimates when including women with a post-diagnostic PA assessment shortly after diagnosis compared to those assessed 1 year after diagnosis, indicating that our findings are likely not due to reverse causation. Moreover, the results did not reveal any differences in the post-diagnostic PA levels across different tumor stages. Women who died before the second follow-up period did not complete both questionnaires. Excluded women who did not completing the second follow-up questionnaire were older at time of breast cancer diagnosis, had a slightly lower alcohol consumption, were more likely to be current smokers, and had a higher proportion of stage I tumors. However, these are not very large differences and we suspect this did not affected the results in a notable manner.

Individual disease management is of great importance to improve long-term outcomes after breast cancer diagnosis. Current international recommendations for PA among breast cancer survivors are equal those for the general population [27–30]. This implies exercising at least 150 min per week, including strength training at least 2 days per week [28, 30]. Although the evidence is incomplete, recommendations can be made to guide cancer survivors in their choices about PA and other modifiable behaviors that may improve long-term outcomes.

## Conclusion

Overall, we observed a dose–response trend in our data for the association between post-diagnostic PA level and both all-cause and breast cancer-specific mortality. We also observed reduced mortality among women with a post-diagnostic PA level ≥5. Our study adds to the knowledge that a modifiable behavior such as PA after breast cancer diagnosis may improve survival. Our results are very promising for women with breast cancer, and indicate that health care professionals should consider adding PA as a part of primary cancer treatment. However, randomized controlled trials are needed to further document this association.

## Additional files

Additional file 1: **Hazard ratios (HR) and 95 % confidence intervals (CIs) of all-cause mortality**^**a**^ **and breast cancer-specific mortality**^**b**^ **according to post-diagnostic physical activity (PA) level and post-diagnostic BMI among 1,327 women from the Norwegian Women and Cancer study, 1991-2011.** (DOCX 13 kb)
Additional file 2: **Hazard Ratios (HR) and 95 % confidence intervals (CIs) of all-cause mortality**^**a**^ **and breast cancer-specific mortality**^**b**^ **according to post-diagnostic physical activity (PA) level by age at diagnosis among 1,327 women from the Norwegian Women and Cancer study, 1991-2011.** (DOCX 13 kb)

## Abbreviations

BMI: Body mass index
CI: Confidence intervals
HEAL: Health, Eating, Activity and Lifestyle study
HR: Hazard ratio
ICD-10: International Statistical Classification of Diseases, Injuries and Causes of Death 10th revision
MET: Metabolic equivalent
NOWAC: The Norwegian Women and Cancer study
P: p-value
PA: Physical activity
SD: Standard deviation
WHI: Women’s Health Initiative Cohort
