# Supplementary material for: Physical activity before and after breast cancer diagnosis and survival - the Norwegian women and cancer cohort study
Source: BMC Cancer. 2015 Dec 16;15:967. doi: 10.1186/s12885-015-1971-9 (PMC4682279; doi:10.1186/s12885-015-1971-9)
Supplement: Additional file 1: — Hazard ratios (HR) and 95 % confidence intervals (CIs) of all-cause mortalitya and breast cancer-specific mortalityb according to post-diagnostic physical activity (PA) level and post-diagnostic BMI among 1,327 women from the Norwegian Women and Cancer study, 1991-2011. (DOCX 13 kb) [file 12885_2015_1971_MOESM1_ESM.docx]

**Additional file 1.** **Hazard ratios (HR) and 95% confidence intervals (CIs) of all-cause mortality^a^ and breast cancer-specific mortality^b^ according to post-diagnostic physical activity (PA) level and post-diagnostic BMI among 1,327 women from the Norwegian Women and Cancer study, 1991-2011**

|  |  | **All-cause mortality** | | |  |
| --- | --- | --- | --- | --- | --- |
| Post-diagnostic  PA level | N deaths | BMI <25  HR (95% CI)^c^ | N deaths | BMI ≥25  HR (95% CI)^c^ | P for homogeneity^d^ |
| 1 | 9 | 4.96 (2.37, 10.40) | 11 | 2.70 (1.33, 5.51) | 0.991 |
| 2 | 24 | 1.30 (0.78, 2.14) | 26 | 1.33 (0.78, 2.29) | 0.444 |
| 3 | 50 | 1.00 (ref) | 30 | 1.00 (ref) | - |
| 4 | 30 | 0.79 (0.49, 1.27) | 10 | 0.71 (0.34, 1.47) | 0.438 |
| 5 | 2 | 0.33 (0.08, 1.39) | 2 | 1.15 (0.27, 4.93) | 0.274 |
| P for trend |  | <0.001 |  | 0.007 |  |
| Breast cancer cases/Total n of deaths | 720/115 |  | 587/79 |  |  |
|  |  | **Breast cancer-specific mortality** | | |  |
| Post-diagnostic  PA level | N deaths | BMI <25  HR (95% CI)^c^ | N deaths | BMI ≥25  HR (95% CI)^c^ | P for homogeneity^d^ |
| 1 | 8 | 5.48 (2.47, 12.17) | 10 | 4.08 (1.83, 9.06) | 0.481 |
| 2 | 18 | 1.15 (0.65, 2.03) | 22 | 1.86 (0.98, 3.54) | 0.158 |
| 3 | 42 | 1.00 (ref) | 18 | 1.00 (ref) | - |
| 4 | 21 | 0.65 (0.38, 1.14) | 10 | 1.22 (0.55, 2.70) | 0.686 |
| 5 | 2 | 0.44 (0.10, 1.66) | 1 | 1.27 (0.17, 9.77) | 0.850 |
| P for trend |  | <0.001 |  | 0.006 |  |
| Breast cancer cases/Total n of deaths | 720/91 |  | 587/61 |  |  |

^a^All causes of death combined.

^b^Breast cancer as cause of death.

^c^Multivariable model adjusted for age and tumor stage at diagnosis, and pre-diagnostic PA level.

^d^Wald’s test for homogeneity.
