# Supplementary material for: Physical activity before and after breast cancer diagnosis and survival - the Norwegian women and cancer cohort study
Source: BMC Cancer. 2015 Dec 16;15:967. doi: 10.1186/s12885-015-1971-9 (PMC4682279; doi:10.1186/s12885-015-1971-9)
Supplement: Additional file 2: — Hazard Ratios (HR) and 95 % confidence intervals (CIs) of all-cause mortalitya and breast cancer-specific mortalityb according to post-diagnostic physical activity (PA) level by age at diagnosis among 1,327 women from the Norwegian Women and Cancer study, 1991-2011. (DOCX 13 kb) [file 12885_2015_1971_MOESM2_ESM.docx]

**Additional file 2.** **Hazard Ratios (HR) and 95% confidence intervals (CIs) of all-cause mortality^a^ and breast cancer-specific mortality^b^ according to post-diagnostic physical activity (PA) level by age at diagnosis among 1,327 women from the Norwegian Women and Cancer study, 1991-2011**

|  |  | **All-cause mortality** | | |  |
| --- | --- | --- | --- | --- | --- |
| Post-diagnostic  PA level | N deaths | 34-49 years  HR (95% CI)^c^ | N deaths | 50-74 years  HR (95% CI)^c^ | P for homogeneity^d^ |
| 1 | 6 | 1.46 (0.61, 3.46) | 14 | 4.33 (2.29, 8.17) | 0.008 |
| 2 | 19 | 1.05 (0.61, 1.82) | 32 | 1.40 (0.86, 2.28) | 0.373 |
| 3 | 43 | 1.00 (ref) | 39 | 1.00 (ref) | - |
| 4 | 21 | 0.63 (0.36, 1.09) | 19 | 0.91 (0.52, 1.59) | 0.805 |
| 5 | 3 | 0.45 (0.13, 1.49) | 1 | 0.36 (0.05, 2.64) | 0.390 |
| P for trend |  | 0.030 |  | <0.001 |  |
| Breast cancer cases /Total n of deaths | 381/92 |  | 946/105 |  |  |
|  |  | **Breast cancer-specific mortality** | | |  |
| Post-diagnostic  PA level | N  deaths | 34-49 years HR (95% CI)^c^ | N deaths | 50-74 years HR (95% CI)^c^ | P for homogeneity^d^ |
| 1 | 6 | 1.67 (0.69, 4.01) | 12 | 5.58 (2.72, 11.43) | 0.003 |
| 2 | 18 | 1.13 (0.64, 2.00) | 23 | 1.45 (0.79, 2.65) | 0.380 |
| 3 | 37 | 1.00 (ref) | 25 | 1.00 (ref) | - |
| 4 | 17 | 0.62 (0.34, 1.14) | 14 | 1.05 (0.54, 2.05) | 0.912 |
| 5 | 2 | 0.39 (0.09, 1.69) | 1 | 0.66 (0.09, 4.95) | 0.745 |
| P for trend |  | 0.020 |  | 0.001 |  |
| Breast cancer cases/Total n deaths | 381/80 |  | 946/75 |  |  |

^a^All causes of death combined.

^b^Breast cancer as cause of death.

^c^Multivariable model adjusted for age, tumor stage at diagnosis, and pre-diagnostic PA level.

^d^Wald’s test for homogeneity.
